# Exacerbation of visual hallucinations in Charles Bonnet syndrome due to the social implications of COVID-19

**DOI:** 10.1136/bmjophth-2020-000670

**Published:** 2021-02-11

**Authors:** Lee Jones, Lara Ditzel-Finn, Judith Potts, Mariya Moosajee

**Affiliations:** 1 Moorfields Eye Hospital NHS Foundation Trust, London, UK; 2 Institute of Ophthalmology, UCL, London, UK; 3 Great Ormond Street Hospital for Children, London, UK; 4 Esme's Umbrella, London, UK; 5 The Francis Crick Institute, London, UK

**Keywords:** COVID-19

## Abstract

**Objective:**

Charles Bonnet syndrome (CBS) occurs secondary to sight loss, characterised by spontaneous visual hallucinations. Symptom manifestation can be influenced by social isolation. This research aims to evaluate the effect of the COVID-19 pandemic lockdown on patients with CBS.

**Methods and analysis:**

A prospective cross-sectional survey of 45 individuals with active CBS. Open and closed ended questions were used to measure patient-reported features of hallucinatory experiences during the COVID-19 lockdown and perceived episode triggers. Analysis comprised of descriptive statistics, analysis of variance and associations, supplemented with qualitative descriptions.

**Results:**

The survey was operational for 31 days during the COVID-19 pandemic (June–July 2020). The mean (±SD) age of respondents was 69.3 (±18) years and the majority (42.2%) had macular disease. Loneliness during the lockdown was associated with changes in the nature of visual hallucinations (p=0.04). Individuals experiencing greater loneliness were, on average, older than those with no changes to their feelings of loneliness (mean age 73.3±17 vs 60.2±19 years; p=0.03). Despite experiencing greater feelings of loneliness (67%), most individuals (60%) had not accessed support services for this reason.

**Conclusions:**

Around half of respondents in this survey experienced exacerbation of visual hallucinations during the COVID-19 pandemic, which may partly be explained by loneliness and/or environmental triggers. We provide suggestions to promote effective patient self-management of symptoms.

**Key messages**

**What is already known about this subject?**

- Visual hallucinations in Charles Bonnet syndrome (CBS) can be a source of significant distress for people with sight loss.

**What are the new findings?**

- Approximately half of people responding to a CBS survey report exacerbation in disease phenomenology during the COVID-19 pandemic, including increased frequency and more problematic visual hallucinations.

**How might these results change the focus of research or clinical practice?**

- Results suggest increasing social interactions, engaging in physical exercise and reducing news exposure may help alleviate symptoms in CBS.
- Findings emphasise the importance of healthcare professionals being aware of CBS risk factors and are conversant with strategies to promote effective patient self-management.

## Introduction

The COVID-19 pandemic has seen countries around the world in a state of mass quarantine (ie, ‘lockdown’) in an attempt to reduce the risk of coronavirus transmission. In the UK, public health measures have recommended people stay at home, restrict visitations and shield vulnerable groups leading to the social isolation of millions.^1^ These measures disproportionately affect certain populations, for example, older adults whose primary social contact is outside of the home, and the visually impaired, many of whom rely on face-to-face or tactile interaction. Negative consequences of social isolation may be felt most by individuals living with Charles Bonnet syndrome (CBS), a condition where social interaction, loneliness and anxiety play a key role in the manifestation of symptoms and individuals’ ability to self-manage the disorder.^2 3^

CBS refers to the presence of visual hallucinations experienced by individuals with reduced vision. Depending on the study, the reported prevalence ranges from 0.4% to 30% among those with sight loss.^4^ Any underlying ocular pathology can precipitate CBS, though literature consistently identifies age-related macular degeneration as a risk factor.^5–7^ The condition is typically associated with older age; however, the pathogenesis of CBS suggests patients of any age may be affected.^6 8^ Typical phenomenology includes transient episodes of simple (ie, shapes and patterns) or complex (ie, life-like scenes, animals and people) hallucinations. Symptoms may persist for many years, with significant detriment to quality of life.^9^ Hallucinations occur without conscious volition and may be triggered by external factors such as trauma and stress.^10 11^ In these circumstances, symptoms may be aggravated and more overwhelming to patients, resulting in a reduced ability to cope and maladaptive behavioural responses.^11^

Evidence suggests visual hallucinations occur as a response to changing sensory input to specific areas of the brain.^12–14^ This theory is supported by studies identifying reduced visual acuity as a risk factor for CBS.^5 15^ Yet, not all visually impaired individuals experience hallucinations, and CBS has been described in those with preserved visual acuity.^8 16 17^ Related theories suggest sensory deprivation as a result of social isolation may be responsible for visual hallucinations. For example, several studies associate living alone with onset of visual hallucinations.^2 18 19^ Due to the theoretical background underpinning social isolation as a potential risk factor in CBS, we sought to explore how the national lockdown in response to the COVID-19 pandemic has affected individuals with CBS. The aim of this study was to investigate hallucinatory episodes of patients with CBS during the lockdown and identify potential triggers for symptom exacerbations and to understand the well-being implications to enable better patient support.

## Materials and methods

In June 2020, an online survey was distributed among members of a campaign group for people living with CBS (Esme’s Umbrella), and charities for people with vision loss (The Macular Society and Retina UK). Patients at Moorfields Eye Hospital National Health Service Foundation Trust reporting CBS symptoms during well-being phone calls with eye clinic liaison officers were also invited to participate. Participants were given the choice of completing the survey online or by telephone with a member of the research team (LD-F). Patient participants were eligible if they had experience of CBS prior to the COVID-19 pandemic, with or without an official diagnosis, and were over the age of 18 years.

**Figure 1:**
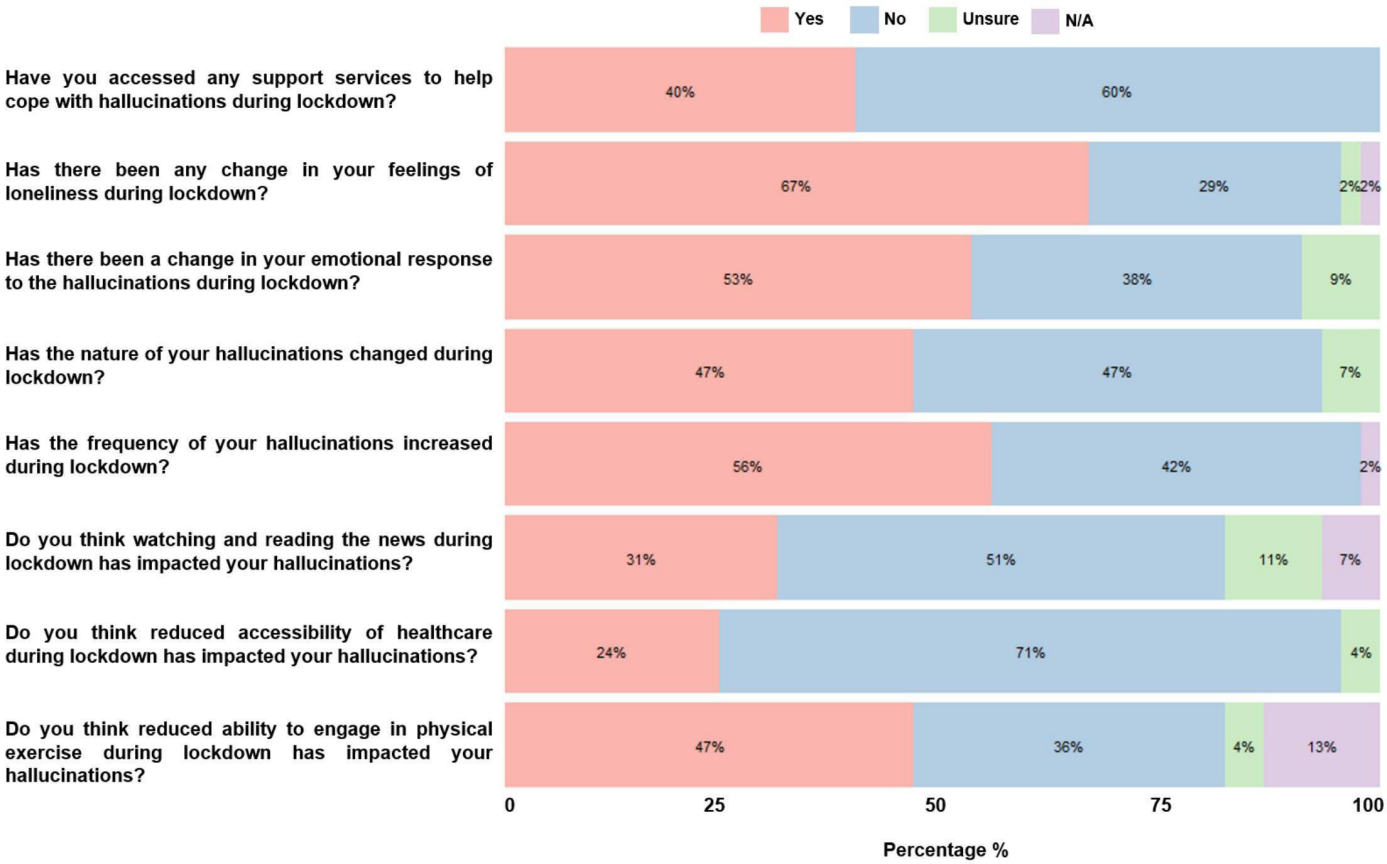
Response distributions for survey items among 45 people with Charles Bonnet syndrome. N/A, not applicable.

The survey was constructed and administered using Qualtrics (Qualtrics, Provo, Utah, USA) and items were designed to measure how visual hallucinations had been affected during the COVID-19 pandemic. There are currently no validated instruments for reporting visual hallucinations in CBS. As such, bespoke survey items were created based on the judgement and expertise of the study authors which consists of two psychologists, an ophthalmologist and the founder of Esme’s Umbrella. The items were developed following consultation with patients with CBS and vision loss charities regarding the nature of complaints from patients with CBS, and was piloted prior to administration. Items consisted of both open and closed ended questions using a Likert scale followed by an optional free text box allowing respondents to provide descriptive information. The survey items are shown in figure 1, which were transposed to allow all responses to be measured and presented on the same scale (ie, ‘Yes’/ ‘No’). The original survey items presented to respondents were structured to allow identification of direction of change in symptoms (ie, increases vs decreases). In addition, the response categories were tailored to fit each item.

Quantitative analysis consisted primarily of descriptive statistics which were supplemented with the qualitative responses. Χ^2^ test of independence was used to determine associations between variables. Kruskal-Wallis tests were used to determine interaction between age and responses.

### Patient and public involvement

A representative from a support group for people with CBS (JP) was involved in the study from the outset. A patient with lived experience of CBS helped to develop the survey questions during a consultation exercise. Specific contributions from both included advice on study design, including defining the aims of the study and feedback on the survey questions.

## Results

There were 45 respondents over 31 days. Mean (SD) age was 69.3 (18) years with 60% being women (n=27) (table 1). Most participants reported having macula-involving disease (42.2%), and 22.2% had multiple ocular diagnoses. A range of hallucinations were reported, complex were most common with the appearance of people or faces (64.4%).

**Table 1:** Respondent demographics, diagnosis and features of visual hallucinations

Category	Subcategory	Count (percentage)
Age range (years)	30–50	8 (17.8)
	51–70	9 (20)
	70+	27 (60)
	Not given	1 (2.2)
Sex	Male	18 (40)
	Female	27 (60)
Principal diagnosis	Macula disease	19 (42.2)
	Optic neuropathy	4 (8.9)
	Retinal dystrophy	6 (13.3)
	Multiple ocular diagnoses (including cataract, pseudoxanthoma elasticum, pathological myopia, microphthalmia)	10 (22.2)
	Other (one case each of sarcoidosis, enucleation and keratoconus)	3 (6.7)
	Not given	3 (6.7)
Living arrangement	Alone	19 (42.2)
	Accompanied	25 (55.6)
	Not given	1 (2.2)
Hallucination characteristics	Geometric shapes	22 (48.9)
	Patterns with/without colour	28 (62.2)
	Flashes of light	22 (48.9)
	Rings or halos	8 (17.8)
	People or faces	29 (64.4)
	Animals/fauna	24 (53.3)
	Plants/flora	20 (44.4)
	Vehicles	12 (26.7)
	Other (including prosopometamorphopsia, palinopsia)	28 (62.2)

As shown in figure 1, 25 respondents (56%) reported increased frequency of visual hallucinations during the pandemic. Changes in the nature of hallucinations and emotional response were reported in 21 (47%) and 24 (53%) respondents, respectively. Participants considered physical exercise to have a protective effect against CBS, where 21 (47%) thought reduced capacity for exercise was responsible for changes in hallucination phenomenology. Almost one-third (31% N=14) of respondents believed that distressing media coverage during the pandemic was responsible for exacerbating hallucination frequency. Thirty (67%) respondents reported greater feelings of loneliness, and there was a statistically significant relationship between loneliness and living alone during the pandemic χ^2^ (1, N=43)=4.35, p=0.04. Despite the changes in disease phenomenology, only 18 (40%) had accessed patient support services through sight loss charities. Free text boxes allowed respondents to provide optional adjunct qualitative detail for each survey item (table 2).

**Table 2:** Example of qualitative responses by survey item

Survey item	Quote description (sex and age range)
Have you accessed any support services to help cope with hallucinations during lockdown?	‘I contacted my GP, I’m now on a waiting list for counselling.’ (Female, 30s)
Has there been any change in your feelings of loneliness during lockdown?	‘I’ve never felt as alone as what I have been feeling during lockdown.’ (Female, 70s) ‘I have found it difficult to talk to family members about the increased hallucinations as not to worry or upset them. I have found I have had to deal with the increased intensity alone.’ (Female, 30s)
Has there been a change in your emotional response to the hallucinations during lockdown?	‘I am now becoming more agitated about seeing them.’ (Male, 80s) ‘I’m more angry with them recently.' (Female, 80s) ‘I have more anxiety about them. The food hallucinations are very disturbing.' (Female, 80s)
Has the nature of your hallucinations changed during lockdown?	‘The hallucinations have become more real and I now argue with my wife that what I see is authentic. I feel I am living in a different house and the people I see are everywhere.’ (Male, 70s) ‘The intensity of the colours and bright lights happens more often, and I see more frightening zombie like faces with blood dripping from the eyes, although all caricature.’ (Female, 30s) ‘They’ve become much more vivid and intense, and they are bigger and move more than before. They are 10× worse and have introduced more abnormal features including people with two heads.’ (Female, 80s)
Has the frequency of your hallucinations increased during lockdown?	‘They used to be 1–2 times a month, they’re now 3–4 days a week.’ (Male, 50s) ‘I’ve found during lockdown my CBS has been around 4 to 5 times per week, when previously it was around 2 to 3 times per week.’ (Male, 30s)
Do you think watching and reading the news during lockdown has impacted your hallucinations?	‘I cut down the amount of times I would tune into the news because in my experience it creates anxiety which triggers more frequent hallucinations.’ (Female, 70s) ‘The news reports over the past months have been stressful and depressing; they’ve caused me significant stress which could correlate with the more frequent than usual hallucinations.' (Male, 30s)
Do you think reduced accessibility of healthcare during lockdown has impacted your hallucinations?	‘I have feelings of anxiety regarding whether or not I will get my injection appointment on time to prevent further damage to my eye.’ (Female, 80s)
Do you think reduced ability to engage in physical exercise during lockdown has impacted your hallucinations?	‘I’m spending more time just sitting, which is when more hallucinations occur.’ (Male, 70s) ‘I’m used to a busy lifestyle. Some days I’m out for up to 12 hours. I think all of a sudden being told to stay at home and only leaving the house for limited reasons played a big factor in more frequent CBS experiences.’ (Male, 30s)

CBS, Charles Bonnet syndrome; GP, general practitioner.

Χ^2^ test of independence showed a statistically significant interaction between loneliness and changes in the nature of hallucinations during the lockdown, χ^2^ (1, N=41)=4.36, p=0.04. As shown in table 2, participants reported larger, more complex, troublesome and disorientating hallucinations, describing greater difficulty distinguishing between hallucinations and reality. Loneliness was however not associated with other items related to CBS phenomenology including increased frequency, χ^2^ (1, N=42)=0.01, p=0.92, or changes in emotional reaction to hallucinations χ^2^ (1, N=39)=0.01, p=0.96.

There was no statistical association between age and changes in hallucination frequency (p=0.55; Kruskal-Wallis test), changes in the nature of hallucinations (p=0.72; Kruskal-Wallis test) or whether respondents had accessed support services (p=0.79; Kruskal-Wallis test). There was a statistically significant association between age and loneliness, whereby those who were lonelier during the pandemic were, on average, older than those with no changes in feelings of loneliness (mean age 73.3±17 vs 60.2±19 years, Kruskal-Wallis test; p=0.03). In addition, those reporting a change in their emotional reaction to hallucinations through the pandemic were, on average, older than those who did not experience an altered emotional response (mean age 73.9±19 vs 65.5±16 years, Kruskal-Wallis test; p=0.03).

## Discussion

People with CBS have experienced changes in visual hallucination phenomenology during the national lockdown associated with the COVID-19 pandemic. There is no established cure for CBS; however, patient education and reassurance play a vital role in improving quality of life.^20^ Treatment pathways in persistent cases include patient counselling and cognitive–behavioural therapy, with a theoretical basis for pharmacological modification of cholinergic, GABAergic, serotonergic or dopaminergic systems, or reduction of cortical excitability.^21^ An important health priority for people with CBS will be to mitigate the impact of the lockdown and subsequent isolation, ensuring they remain socially connected. The challenge will be to ensure all those struggling with visual hallucinations are promptly identified through effective surveillance and signposted to appropriate social support.

Loneliness has been a significant and longstanding societal challenge, which has been amplified by the COVID-19 pandemic.^22 23^ A recent study found that loneliness was considered the most concerning aspect of the pandemic among the general population, more so than contracting the virus itself.^24^ Our results show that people who were lonelier during the lockdown were, on average, statistically significantly older than those reporting no change in loneliness (mean age 73.3±17 vs 60.2±19 years, respectively). This is perhaps unsurprising given the well-established association between loneliness and ageing.^25^ There was also evidence that respondents who had become more fearful and agitated by their hallucinations were, on average, statistically significantly older (mean age 73.9±19 years) compared with those without changes in their emotional reaction (mean age 65.5±16 years), which may partly be explained by increased loneliness. Circumstances in which poor health outcomes may be particularly acute occur when people find themselves unexpectedly alone, such as death of a spouse or partner and separation from friends and family; conditions which have become highly prevalent during the COVID-19 pandemic. The biological response to loneliness is also significant, for example, alterations in the immune system cellular mechanisms contribute to a weakened immune response, suggesting lonely individuals become more vulnerable to infectious diseases.^26^

Our analysis highlighted that self-reported loneliness was associated with a change in the nature of the visual hallucinations experienced during the lockdown. For example, many respondents described how prior to the pandemic their hallucinations were typically unobtrusive and small in size. Lilliputian hallucinations are visual phenomena in which the hallucinations are miniature in size. During the lockdown these hallucinations had grown to become larger and more difficult to ignore, such as the arrival of a full-sized person standing in the same room. Similarly, in some cases hallucinations were no longer static and now featured movement. For example, one participant described being followed by a shadowed figure whenever they were walking, while others described feelings of claustrophobia due to being trapped by encroaching plants and foliage. This finding supports previous theories that sensory deprivation, as a result of social isolation, is associated with visual hallucinations in CBS.^2 19 20^ However, living conditions do not fully explain hallucination phenomenology as previous findings identify a higher prevalence of CBS among married individuals as well as those cohabiting.^27^ Our results provide convincing evidence for a link between loneliness and the nature of hallucinations presented in CBS. However, we did not observe a statistically significant association between loneliness and hallucination frequency or emotional reaction to hallucinations. One explanation for this could be the way in which the survey items were constructed, as the items intended to measure subjective changes in hallucination phenomenology. Yet if hallucinations were already highly prevalent and continuous prior to the pandemic which persisted throughout the lockdown, then it would be expected that no increase in frequency would be perceived or reported.

Social support is often described as the most powerful remedy for loneliness,^28^ and thus plays a critical role in determining patient health outcomes. For example, a high degree of social support is generally associated with lower health symptomatology and better mental and physical health.^29 30^ People with greater social and emotional support in their lives tend to be healthier than those who are lacking in support.^31^ Prolonged isolation for those who encountered the lockdown alone may contribute to an increase in psychological distress due to reduced exposure to sources of support (eg, family and friends) and increasing the importance of personal resources such as self-efficacy and resilience.^32^ Indeed, we found that those living alone reported greater feelings of loneliness during the lockdown. Additionally, we identified age as a risk factor for loneliness. This finding contrasts with results from similar studies investigating loneliness during the COVID-19 pandemic, whereby an inverse association between age and loneliness has been reported.^32–34^ Such results could be due to older adults typically displaying a lower reactivity to stress^35^ and exercising greater resilience,^36^ while having more experience with being alone and with life-threatening medical situations.^32^ The association between age and loneliness identified in our study could be explained by a difference in the needs of visually impaired older adults compared with community-dwelling older adults without vision impairment. This outcome raises important questions regarding the manifestation of loneliness among the visually impaired and signals the need for strategies aimed to promote social participation and accessibility. In the UK, interventions to facilitate sociable contact with people with sight loss during the pandemic have been implemented, such as well-being phone calls to vulnerable populations, and charitable organisations dispatching remote counselling services, which may bolster psychological well-being and allay feelings of isolation.^37^ Our findings provide useful data about loneliness among the visually impaired during the COVID-19 pandemic, which may help maximise the effectiveness of targeted charity and hospital-based social support services. Yet it is critical to consider other factors that put individuals at risk of loneliness, not merely based on age, in order to effectively identify those who would benefit from intervention. Moreover, patients with CBS are often averse to disclosing symptoms due to fears that the hallucinations are indicative of cognitive impairment,^38^ therefore tactful inquiry as to the presence of visual hallucinations is essential when operating both remote and face-to-face ophthalmic services.

A number of behavioural strategies may provide relief during hallucinatory episodes, including adjusting lighting and practising specific eye movements.^39^ Though limited, these interventions are based on anecdotes from patients and medical experts and are simple to implement, are of no cost and can be applied immediately. We provide the full toolkit in box 1 which may serve as a clinically useful resource for clinicians when consulting with patients about self-managing visual hallucinations.

Box 1 **Techniques for minimising or eliminating visual hallucinations**

- When the hallucinations start, look from right to left once every 15 s without moving your head.
- Try to touch the hallucination.
- Stare straight at the hallucination.
- Turn your head to alternative sides, then move the head towards each shoulder in turn.
- Walk around the room or to another room.
- Shine a torch from below your chin in front of (not into) your eyes.
- Change the light level in your room or the activity you are doing.

It is noteworthy that our data suggest many individuals were seemingly unperturbed by the lockdown, and a high proportion did not experience notable changes in their visual hallucinations. For example, 38% of respondents believed their emotional reaction to hallucinations was unchanged. One explanation for this could be habituation to CBS over time, as long-term or refractory cases may be better equipped with coping mechanisms or may possess greater resilience to hallucinations. While all respondents had experience of CBS prior to the lockdown, we did not capture details on individual CBS time frames and so cannot make further comparisons. Previous research has shown a lack of association between overall duration of CBS and negative outcome,^9^ suggesting patients may better tolerate hallucinations or adjust to their occurrence over time. It is also striking that, for example, 71% did not believe reduced access to healthcare during the lockdown impacted their CBS. This may be due to the strengthening of remote consultations or could indicate the success of public health messaging throughout the pandemic that hospitals remain open despite travel restrictions, and that people should not ignore symptoms that might require urgent medical attention.

In this survey, 31% of respondents attributed negative or upsetting news stories to increased frequency and onset of more distressing hallucinations. Evidence suggests ‘headline stress disorder’, a high emotional response to ongoing media coverage, may lead to physical dysfunction and progression of underlying conditions.^40^ Those working in eye care must consider the impact of repeated distressing media consumption and understand its potential role in amplifying CBS symptoms. One management solution is to advise patients to stay informed via authoritative news sources, and to be prudent and critical about unofficial reports. For example, sensationalised news coverage from unauthorised sources during the 2014 Ebola outbreak is believed to have propelled avoidable stress and panic among the general public.^40^ While two-thirds of our respondents did not believe media coverage during the pandemic contributed to their visual hallucinations, a significant minority (31%) suspected a casual effect. Individual personal characteristics may explain this difference, as some individuals may be more vulnerable to news-related stress while others may gain comfort from feeling well informed. Nevertheless, we speculate that encouraging patients’ critical thinking skills may minimise risk of misinformation, which in turn could have clinically relevant benefits such as reduced stress and may help to harness positive attributes of traditional media.^41^

Physical exercise is a well-established effective therapy for many chronic conditions.^42^ In this survey, 47% of respondents believed reduced engagement in physical exercise was responsible for changes in hallucination phenomenology. Tackling prolonged sedentary behaviour through sustainable home exercise programmes will help counter patient’s inactivity, which is a known risk factor in CBS. Given the broad range of ages observed in our sample (30–94 years), and likely large variability in clinically measured visual functioning, specific modality, frequency, volume and intensity of exercise will be dependent on individual capability. Healthcare professionals may wish to consider recommending suitable home exercise regimens for people with CBS at times where outdoor mobility cannot easily be achieved.

This study has certain limitations. Our sample consisted of self-selecting participants which may have incurred a sampling bias, whereby those with strong opinions on the study matter may be more likely to complete the survey. In addition, survey methods may be prone to response bias. However, the survey was confidential, thus limiting the impact of such tendencies. We used only single items for each study domain. For example, when measuring loneliness, we relied on a single and direct self-labelling item. While this is a common means of measuring loneliness in epidemiological studies, there are important shortcomings such as the willingness of respondents to admit to being lonely due to the associated stigma. Direct assessment does not account for possible unawareness of loneliness and relies on the conscious recognition and verbalisation of feeling lonely, which may result in under-representation.^43^ Future studies in this area using alternative methods for indirectly measuring loneliness, such as the revised UCLA Loneliness Scale,^44^ would be valuable for comparison of results. There is a need for further research to establish risk factors associated with CBS onset and a greater understanding of environmental triggers and circumstances that intensify symptoms in CBS.

We are yet to understand the long-term consequences of the drastic lifestyle changes caused by the COVID-19 pandemic. Widespread isolation of vulnerable populations will likely persist throughout the rollout of vaccination programmes, providing unique circumstances that may lead to exacerbation or new onset of CBS. As we emerge from this pandemic, it will remain essential that all clinicians working in the ophthalmic sector are aware of the difficulties faced by individuals with CBS and be conversant with available management strategies while continuing to promote good mental health.
